# Mappability and read length

**DOI:** 10.3389/fgene.2014.00381

**Published:** 2014-11-10

**Authors:** Wentian Li, Jan Freudenberg

**Affiliations:** The Robert S. Boas Center for Genomics and Human Genetics, The Feinstein Institute for Medical Research, North Shore LIJ Health SystemManhasset, NY, USA

**Keywords:** next-generation sequencing, repeats, mappability, power-law distribution, copy number variations

## Abstract

Power-law distributions are the main functional form for the distribution of repeat size and repeat copy number in the human genome. When the genome is broken into fragments for sequencing, the limited size of fragments and reads may prevent an unique alignment of repeat sequences to the reference sequence. Repeats in the human genome can be as long as 10^4^ bases, or 10^5^ − 10^6^ bases when allowing for mismatches between repeat units. Sequence reads from these regions are therefore unmappable when the read length is in the range of 10^3^ bases. With a read length of 1000 bases, slightly more than 1% of the assembled genome, and slightly less than 1% of the 1 kb reads, are unmappable, excluding the unassembled portion of the human genome (8% in GRCh37/hg19). The slow decay (long tail) of the power-law function implies a diminishing return in converting unmappable regions/reads to become mappable with the increase of the read length, with the understanding that increasing read length will always move toward the direction of 100% mappability.

## 1. Introduction

Shotgun and next-generation sequencing (NGS) involve shredding the genome into smaller fragments, and sequence either full or part of the fragments. The sequenced fragments are called reads. Overlapping of sequences between reads are the basis of *de novo* assembly (Scheibye-Alsing et al., 2009). Reference assembly is based on mapping reads to a reference genome. The task of reference assembly is straightforward when the read length is long enough. Despite the theoretical possibility that a sequence can be free of any repeats at a specific length scale *k* (the De Bruijn sequence, Ralston, 1982), real genomes such as the human genome contain many repetitive sequences. Therefore, length-*k* reads may not be mapped uniquely. The regions where these reads are originally derived are defined as the “unmappable regions” at the read length *k*, and these reads are defined as “unmappable reads.”

**Figure 1:**
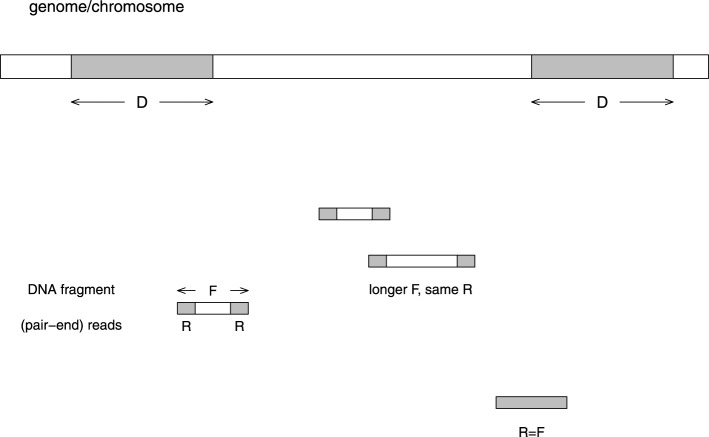
**Illustration of the problem caused by repeats in the reads alignment to a reference genome**. The length of the repeat unit is *D* basepairs, the length of a (typical) DNA fragment is *F*, and the length of a read in the paired-end sequencing is *R*. If *D* < *R*, the fragment can be mapped to the genome uniquely. On the other hand, if *D* > *F*, the fragment is unmappable. If *R* < *D* < *F*, the fragment may or may not be mappable. When the whole fragment is sequenced, we consider *R* = *F*. Usually the fragment size is not fixed, whereas the read length is fixed. The distribution of fragment sizes and read lengths are *P_F_* = *P*(*x* = *F*) and *P_R_* = *P*(*x* = *R*). The distribution of maximum repeat length and copy number is *P_D,C_* = *P*(*x* = *D,y* = *C*). The distribution of fixed repeat length and copy number is *P*_*D*_0_,*C*_ = *P*(*x* = *D*_0_, *y* = *C*).

Figure 1 illustrates various factors which influence mappability. (1) The distribution of fragment size *F* (*P_F_*). This distribution clearly depends on the way fragments are sheared. (2) The distribution of the size of reads *R* (*P_R_*), which does not contain the pairing information between the two reads from the same fragment. (3) The distribution of repeats, in both the repeat length (*D*) and the number of copies (*C*). Note that for read-alignment purpose, both the direct and the reverse complement strands should be considered, e.g., *aaagg* and *ccttt* are repeats. There are two approaches in counting repeats: (3a) Only “maximal repeats” are considered (Gusfield, 1997). In this definition, if the length is increased by one to D+1 (extending either to left or right), there is no longer a repeat. (3b) Fixed length (*D*_0_) repeats may or may not be “maximal.” For example, a *D*_0_-length repeat may be part of a larger repeating unit. This definition is more relevant to the situation where the read length is fixed. The two distributions are *P_D,C_* and *P*_*D*_0_,*C*_.

Other considerations further complicate the situation: (4) Given *P_F_, R, P_D,C_*, the zero-mismatch mappability problem might be discussed rigorously. However, most of the alignment programs allow mismatches, and we have a new distribution *P_D,C,M_* = *P*(*x* = *D*, *y* = *C*, *z* ≤ *M*), where *M* is the maximum number of mismatches allowed. Take *M* = 1 for example, the appearance of *aaagg* and *cctat*, for example, contributes to the distribution at *D* = 5, *C* = 2, and *M* = 1. (5) The existence of copy number variations (CNV) (Pinkel et al., 1998) implies that the person's genome may not be the same as the reference genome, which is used by everybody for alignments. In a sense, the reference genome is not unique: there are many versions corresponding to different forms of CNV.

Obtaining empirically the redundancy distribution *P_D,C,M_* is computationally difficult for a large genome. Our previous work is limited to the situation of zero-mismatch (*M* = 0) and fixed repeat length *D*_0_ (Li et al., 2014). We also assume that whole fragments are sequenced (*F* = *R*). Using the *k*-mer from the sequenced/assembled portion of the human genome (GRCh37/hg19) as surrogate of reads with length *R* = *D*_0_, we obtain the fixed-length distribution *P*(*x* = *D*_0_, *y* = *C, z* = 0) (Li et al., 2014). *P*(*C* = 1) is the proportion of uniquely mapped reads. We will review the results obtained in the analysis and discuss various results.

## 2. Unassembled portions of the human reference genome

We first examine the unsequenced/unassembled region of the human genome. Because reads from these regions are often available, these are unassembled rather than “unsequenced” (Rudd and Willard, 2004). There are four types of unassembled regions: (1) telomeres; (2) centromeres; (3) short-arms of acrocentric chromosomes (chr13,14,15,21,22, Y); and (4) large heterochromatic regions (in chr1,9,16, Y). All these regions involve repeat sequences.

The telomere regions contain simple repeats of the hexamer *ttaggg/ccctaa* (Moyzis et al., 1988). This motif is closely related to the telomeric sequence in other genomes (Blackburn et al., 2006). Specifying telomere sequence in a reference genome is difficult because repeat length varies with the age (Blasco, 2005). The G+C content of the above hexamer is 50%, which is higher than the genome-wide value of 40% (Li, 2013). The subtelomeric sequence is also highly repetitive (Riethman et al., 2004), with more varieties in repeat length and pattern, which result from ancestral duplications (Ambrosini et al., 2007; Churikov and Price, 2008).

The centromere region (Willard, 1990; Aldrup-MacDonald and Sullivan, 2014) consists of alpha satellite DNA with 171 basepairs (Manuelidis, 1976, 1978; Vissel and Choo, 1987). In the alpha satellite, the strand symmetry (see e.g., Li, 1997) is reasonably preserved for C% (18.8%) ≈ G% (19.4%), though less so for T% (32.9%) and A% (28.8%). The dimers AG/CT, TT/AA, CA/TG, GA/TC are over-represented in the alpha satellite, and AT, TA, CC/GG, CG, GT/AC under-represented. These dimer frequencies can also be modeled by a Markov chain (Cocho et al., 2014). New computational efforts to fill the assembled centromeres led to a great reduction of N's in GRCh38 (Miga et al., 2014).

The short-arms of acrocentric chromosomes consist of four well partitioned cytogenetic features (from p-term toward the centromere): satellite, stalk, short-arm, and centromere (Wyandt and Tonk, 2011). The repeat composition in these arms are more complicated, with some mainly consisting of HSat2,HSat3, other consisting of HSat1, beta, and gamma satellites. The large heterochromatin regions, such as those on chromosomes 1,9,16, contain similar satellite repeat sequences (Jones, 1973; Jones and Prosser, 1973). These regions are mainly composed of HSat2 and HSat3 evolving from the ancestral pentamer *cattc/gaatg* (which can also be written as attcc/ggaat, Gredy et al., 1992). In a renewed effort, subfamilies of HSat2/HSat3 are identified and cataloged into a database (Altemose et al., 2014).

The amount and proportion of N's (unassembled bases) in the human reference genome (GRCh37 and GRCh38, from Genome Reference Consortium) is 234 Mb or 7.6% and 151 Mb or 4.9%. There is a contrasting difference of the proportion between metacentric and acrocentric chromosomes (4.3 vs. 25.3% in GRCh37, and 2.0 vs. 20.8% in GRCh38). If we exclude chromosomes 1, 9, and 16 with the large heterochromatin regions, the rest of 14 metacentric autosomes achieve a rather low unsequenced rate of 2.6% in GRCh37 and 0.3% in GRCh38.

Since most unassembled regions contain short tandem repeats, the chance for a read from other regions to be aligned to these regions is relatively small. In fact, reads from unassembled regions can be identified as being distinct by their *k*-mer composition (Macas et al., 2010; Koch et al., 2014). However, this does not prevent mis-alignments within these regions. The better understanding of the sequence of these regions may subsequently help to develop methods that can determine repeat length variations, such as telomere length, from the read data.

## 3. Length distribution of DNA fragments and reads

Long DNA sequences are broken into smaller DNA fragments by various means (Quail, 2010), such as sonication and nebulization. Whatever the fragmentation method, the sizes of the fragments in the DNA library is an important parameter (Head et al., 2014). The fragment size distribution is usually single-peaked with the typical size appropriate for the subsequent sequencing methods.

Unlike the fragment size, read size is precisely specified for most sequencing methods. Many companies use pair-end sequencing of relatively short read lengths (2 × 35 bp for Complete Genomics, 2 × 50 for SOLiD of Life Technology/Applied Biosystems, up to 2 × 300 for Illumina). The Ion Torrent of Life Technologies and 454 of Rouche have longer read lengths, up to 400 and 1000 bases, respectively. In comparison, Sanger sequencing can handle up to 1000 bases DNA fragments.

The Pacific Biosciences' single-molecule real-time (SMRT) sequencing (Eid et al., 2009; Roberts et al., 2013) is not equivalent to the highly parallel NGS. However, it is an approach that can produce much longer reads and it may not even need a library preparation (Coupland et al., 2012). For SMRT, *P_R_* instead of *P_F_* is more relevant. Sequences produced by the different technologies are still not 100% identical due to sequencing errors (Huddleston et al., 2014).

## 4. Distribution of exact repeats in the human reference genome

Let's use a simple sequence to illustrate the difference between *P_D,C_* where D is the length of maximal repeats, and *P*_*D*_0_, *C*_ where *D*_0_ is a fixed length: *atcgaaatatccatcc* (reverse complement *ggatggatatatttcgat*). There is one maximal repeating tetramer, *atcc/ggat* (*D* = 4, *C* = 2), and one maximal repeating trimer, *atc* (*D* = 3, *C* = 3). We include *atc* but not *tcc* as another repeat unit because there is an extra copy of *atc* which is not part of *atcc*. For the same reason, *at*, *tc* are independent repeating dimers (*D* = 2, *C* = 4), but not *cc*. On the other hand, with the fixed length *D*_0_ = 3, there are four repeating trimers, *atc/gat* (*C* = 3), *cga/tcg*, *ata/tat*, and *tcc/gga* (*C* = 2). Three of them are part of larger repeat unit of length *D*_0_ = 4.

Obtaining *P_D,C_* for the reference genome needs a pre-processing of the sequence by a suffix array (Manber and Myers, 1993; Crochemore et al., 2007) or other similar data structures (Berger et al., 2013), such as Burrows-Wheeler transform (Burrows and Wheeler, 1994) or FM-index (Ferragina and Manzini, 2005). It is of crucial importance to have a memory(space)-efficient algorithm, as the human genome size is 3 Gbase (or 6 Gbase considering reverse complement) and a typical computer nowadays has only a few Gbyte memory. Compared to suffix tree (Gusfield, 1997), suffix array is known to be more space-efficient. Thus, for genome scale repeat analysis, suffix array is preferred over suffix tree (Sadakane and Shibuya, 2001; Hon et al., 2004; Becher et al., 2009; Barenbaum et al., 2013).

The *P*_*D*_0_,*C*_ distribution is more relevant to the read set, and comparatively easier to obtain. However, the relationship between *P_D,C_* and *P*_*D*_0_,*C*_ may not be trivial. From *P*_*D*_0_,*C*_ to *P_D,C_*, one may first determine the histogram *P*_*D*_0_,*C*_ at *D*_0_ and *D*_0_ + 1, then remove the type counts at *D*_0_ that are part of repeating (*D*_0_ + 1)-mer type. In practice, the situation can be complicated as one (*D*_0_ + 1)-mer may contribute two *D*_0_-mer types. Without more detailed information of the repeating pattern, subtracting *N*_*D*_0_ + 1_ from *N*_*D*_0__ is the least one could do, as it provides an upper limit to *P_D_*.

**Figure 2:**
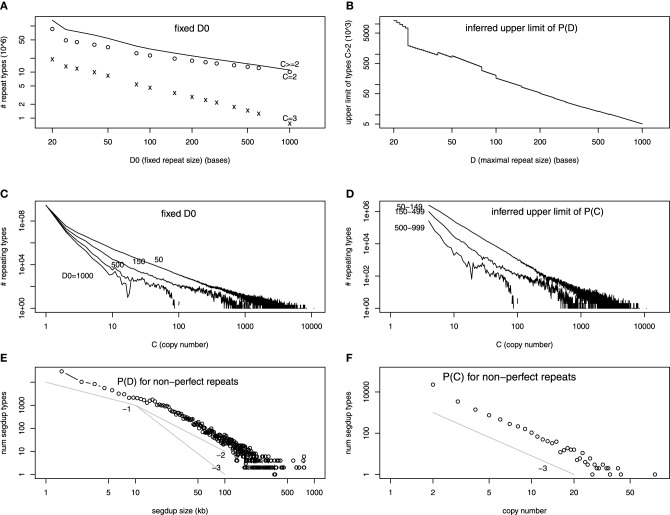
**(A–D)** are on exact repeats and **(E–F)** on approximate repeats, all in log-log scale. **(A)** The number of repeat types as a function of the fixed repeat unit length *D*_0_. The number of repeat types with exact two (three) copies in the genome, *C* = 2 (*C* = 3), are shown separately. **(B)** The difference of number of repeat types at *D*_0_ and *D*_0_ + 1. This is an upper limit of the number of maximal repeat types at *D*. **(C)** The number of repeat types at fixed repeat unit lengths (*D*_0_ = 50, 150, 500, 1000, as a function of copy number C). **(D)** The difference between the number of repeat types at different *D*_0_'s (e.g., between *D*_0_ = 50 and *D*_0_ = 150). This represents the sum of upper limits of number of maximal repeat types at length *D*, summing over all *D*'s between the two values (e.g., 50 and 150). **(E)** Number of appearence in the segmental duplication track from the UCSC Genome Browser with certain size *D* as a function of *D*. The three power-law functions, 1/*D*, 1/*D*^2^, 1/*D*^3^ are drawn for a comparison. **(F)** Number of segmental duplication names as a function of copy number *C* (number of pairwise alignment lines plus one). The power-law function 1/*C*^3^ is drawn for a comparison.

The number of repeat unit types at fixed *D*_0_ in the assembled human reference genome has been obtained at various *D*_0_'s (Li et al., 2014). Most of these repeat types only occur in the genome twice (*C* = 2). The number of repeat types for *C* = 2, *C* = 3, and *C* > 2 is plotted as a function of *D*_0_ in Figure 2A (in log-log scale). The fact that it is almost a straight line indicates that the decay is a power-law, which is a widespread distribution in nature (Sornette, 2006). We extrapolate *N*_*D*_0__ at all *D*_0_'s between 20 and 1000, using a power-law relationship between two neighboring points in Figure 2A (or linear relationship in log-log scale). Subtracting *N*_*D*_0_ + 1_ from *N*_*D*_0__, we infer the upper limit of *P_D_* in Figure 2B.

The copy number information is ignored in Figure 2B. The *P_C_* when *D*_0_ is fixed is shown in Li et al. (2014) which is reproduced in Figure 2C. If we subtract *N*_*D*_0_ = 150,*C*_ from *N*_*D*_0_ = 50,C_, it will sum up the upper limit of all *N_D_* for 50 < *D* < 150. It is done in Figure 2D. Figures 2B,D provide evidence that *P_D,C_* is a power-law function in both repeat unit length and copy number.

## 5. Distribution of approximate repeats in the human reference genome

The distribution *P_D,C,M_* or *P*_*D*_0_,*C,M*_ allowing up to *M* mismatches is much harder to obtain due to computational constraints (Derrien et al., 2012). Take our toy sequence for example: *atcgaaatatccatcc/ggatggatatatttcgat*: with mismatch *M* = 1, there are three clusters of pentamers (*D* = 5) that repeat, with 3, 2, and 4 pentamer types, respectively. A pentamer in a cluster should be less than or equal to *M* mutation away from pentamers in other clusters. But it is not necessary that any two pentamers should be *M* or less mutations away. Since one-mutation path can link all *D*_0_-mers, they consist of one huge cluster. In a real sequence with limited length, however, the genome cannot sample all possible *D*_0_-mers, breaking the path to separate *D*_0_-mer types into clusters.

We use the segmental duplication track (SegDup) in the UCSC Genome Browser to examine the length and copy number distribution for approximate repeats. SegDup was obtained by aligning RepeatMasker (http://www.repeatmasker.org) filtered 400 kb fragments to the reference genome by BLAST (http://blast.ncbi.nlm.nih.gov) (Bailey et al., 2001). The BLAST alignment result is extended to obtain approximate maximal repeats. The minimum length of the SegDup is 1 kb, and the condition of >90% identity in the pairwise alignment is imposed.

Figure 2E shows the frequency of SegDup with certain sizes appear in the track, as a function of the size. Figure 2F is the frequency of SegDup labels, as a function of *C*. Since Figures 2E,F are in log-log scale, we have shown that repeats with mismatches have power-law distribution for both *D* and *C*. This power-law distribution for size is consistent with other studies: the self-alignment for smaller genomes shows similar power-law like distribution in Gao and Miller (2011, 2014). We also draw power-law functions with the known exponents: 1/*D*, 1/*D*^2^, and 1/*D*^3^ for size distribution, and 1/C^3^ for copy number distribution. The size distribution seems to follow 1/*D* for smaller sizes, whereas 1/*D^a^* (2 < *a* < 3) for larger SegDup regions. The copy number distribution is clearly 1/*C*^3^. Interestingly, the 1/*D*^3^ function is predicted by a neutral duplication dynamics model (Massip and Arndt, 2013).

## 6. Proportion of unmappable reads as a function of read length

The unmappability rate depends on whether it is viewed from the reads or the reference sequence perspective. Take the example of our toy sequence, *atcgaaatatccatcc*: of the 13 tetramer counts, two are copies of *atcc*. The proportion of reads counts that are unmappable at *k* = 4 is then 2/13 = 15%. However, the two copies of *atcc* cover 8 base positions, so the proportion of unmappable regions is 8/16 = 50%.

With *k*-mer count information but no locations, only the first proportion can be calculated. The number of read counts which are unmappable in the assembled portion of the human genome is shown in Figure 1 of Li et al. (2014). The proportion of reads that are unmappable to the assembled portion of the human genome is 28.4, 20, 16.2, 11.3, 8.2, 4.3, 3.4, 2.4, 2, 1.7, 1.5, 1.3, 1.2, 1.1, and 0.8% at read lengths of 20, 25, 30, 40, 50, 80, 100, 150, 200, 250, 300, 400, 500, 600, and 1000. The fall of these proportions is faster (judged by the slope of the straight line in log-log scale) when the read length is shorter than 80–100, slower when the read length is longer. This led to the “diminishing return” with the read length in Li et al. (2014).

To evaluate unmappable regions in the genome, the location of the unmappable reads should be known. We have carried out an alignment for the length-1000 unmappable reads (Li and Freudenberg, 2014). The unmappable regions at the 1000-bp level cover a size of 35 Mb, or around 1.2% of the assembled portion of the human genome, larger than the 0.8% from the perspective of read population.

## 7. Discussion

The central thesis of this paper is that if the sequencing produces shorter reads, the length of any repeat unit in the genome sets an upper limit on mappability (a concept applicable to both the read and to the chromosome region). The distribution of repeat lengths, of fragment sizes (if a paired-end method is used), and of read length, together determine the proportion of genome that can be aligned/mapped.

In an analysis (Becher et al., 2009), a repeat of 67632 bases (*C* = 2) is identified in the human genome, with both copies in chromosome 1. The longest repeat that appears in two different chromosomes has length 21864, appearing in chromosomes 1 and 5. This study did not consider the reverse complement strand, thus it leaves the possibility of finding even longer repeat lengths. For other genomes, long repeat lengths have been reported, such as a 41 kb repeat in *E. coli* (Haubold and Wiehe, 2006).

If mismatch is introduced, the repeat (duplication) size can be even larger. Tandem repeats of 38.8 kb (chr1), 23.6 kb (chrY), 22.9 kb (chr17), are listed in Warburton et al. (2008). Up to 200 kb segmentally duplicated regions are examined in Zhang et al. (2005). On Y chromosome, the largest duplication length is 1.5 Mb (Sainz et al., 2006). A 106 kb tandem repeat and CNV within the repeat is reported to be associated with male infertility (Avidan et al., 2003). In the SegDup track from the UCSC Genome Browser, duplications of sizes of 400 kb appear on chromosomes 9 and 10.

The repeat-caused unmappable regions are not only problematic for achieving 100% sequencing, but also, by their tendency to cause genomic instability, casts doubt on the concept of a reference genome. Even the simplest tandem repeats are shown to be under-counted in the reference genome, exhibit high level of CNV (Sharp et al., 2005), affect related gene expression (Stranger et al., 2007), and introduce heterochromatin, which silences nearby genes (Brahmachary et al., 2014). Typing CNVs is the goal of many NGS applications in human complex diseases study, forensics, disease markers (Budowle et al., 2009; Zhang et al., 2009; Bassett et al., 2010; Girirajan et al., 2011), but one should keep in mind the uncertainty of the repeat regions in the reference genome, which are prone to CNV.

Can we equate unmappability to being biologically less important? First, short repeats, which are well known to be disease-causing (La Spada and Taylor, 2010), may expand to longer enough repeat segments that are unmappable. Secondly, genes do exist in repeat regions. The gene *TPTE* was found on the acrocentric arm of chromosome 21 (Chen et al., 1999; Guipponi et al., 2000; Eichler et al., 2004). Many RefGenes are located in the 1 kb-unmappable regions in the assembled reference human genome (Li and Freudenberg, 2014; Li et al., 2014). Thirdly, repeats or duplications are the raw material for evolution (Ohno, 1970). As an anecdotal evidence, all immunoglobulin genes are located near centromeres or telomeres which are full of repeats. To summarize, we have enough facts to conclude that repeats and unmappable regions should not be ignored for a comprehensive analysis of the human genome.

### Conflict of interest statement

The authors declare that the research was conducted in the absence of any commercial or financial relationships that could be construed as a potential conflict of interest.
